# Action observation produces motor resonance in Parkinson's disease

**DOI:** 10.1111/jnp.12133

**Published:** 2017-09-11

**Authors:** Judith Bek, Emma Gowen, Stefan Vogt, Trevor Crawford, Ellen Poliakoff

**Affiliations:** ^1^ Division of Neuroscience and Experimental Psychology School of Biological Sciences Faculty of Biology Medicine and Health Manchester Academic Health Science Centre University of Manchester UK; ^2^ Department of Psychology Lancaster University UK

**Keywords:** Parkinson's disease, motor resonance, visuomotor priming, action observation

## Abstract

Observation of movement activates the observer's own motor system, influencing the performance of actions and facilitating social interaction. This motor resonance is demonstrated behaviourally through visuomotor priming, whereby response latencies are influenced by the compatibility between an intended action and an observed (task‐irrelevant) action. The impact of movement disorders such as Parkinson's disease (PD) on motor resonance is unclear, as previous studies of visuomotor priming have not separated imitative compatibility (specific to human movement) from general stimulus‐response compatibility effects. We examined visuomotor priming in 23 participants with mild‐to‐moderate PD and 24 healthy older adults, using a task that pitted imitative compatibility against general stimulus‐response compatibility. Participants made a key press after observing a task‐irrelevant moving human finger or rectangle that was either compatible or incompatible with their response. Imitative compatibility effects, rather than general stimulus‐response compatibility effects, were found specifically for the human finger. Moreover, imitative compatibility effects did not differ between groups, indicating intact motor resonance in the PD group. These findings constitute the first unambiguous demonstration of imitative priming in both PD and healthy ageing, and have implications for therapeutic techniques to facilitate action, as well as the understanding of social cognition in PD.

## Background

Observing the actions of others increases motor learning (e.g., Stefan, Classen, Celnik, & Cohen, 2008; Vogt *et al*., 2007), as well as facilitating social interaction and understanding (e.g., Chartrand & Bargh, 1999; Iacoboni *et al*., 2005). Importantly, movement can be influenced even by observation of a task‐irrelevant stimulus; this is demonstrated behaviourally through visuomotor priming (also termed ‘automatic imitation’) based on compatibility between observed and executed actions (e.g., Brass, Bekkering, & Prinz, 2001; Gowen, Bradshaw, Galpin, Lawrence, & Poliakoff, 2010). For example, a key press response is facilitated by observing a downward finger movement, but slowed by viewing an upward movement. Compatibility effects, determined by subtracting reaction times for compatible actions from those for incompatible actions, are typically greater for human movement than for non‐biological movement (see Gowen & Poliakoff, 2012, for review). It is thus inferred that compatibility effects from human movement (herein referred to as *imitative compatibility*) reflect simulation of the observed action, or *motor resonance* (e.g., Uithol, van Rooij, Bekkering, & Haselager, 2011), rather than general stimulus‐response mechanisms. Motor resonance is subserved by a frontoparietal mirror neuron system, identified in humans through neuroimaging and electrophysiological studies, as part of a broader action observation network (Caspers, Zilles, Laird, & Eickhoff, 2010; Rizzolatti, Cattaneo, Fabbri‐Destro, & Rozzi, 2014). The basal ganglia have also been suggested to be involved in action observation, based on neuroimaging evidence from healthy individuals (Kessler *et al*., 2006) and subthalamic nucleus recordings in Parkinson's disease (PD; Alegre *et al*., 2010; Marceglia *et al*., 2009). It is thus important to consider the impact of neurological movement disorders such as PD on the internal representation of action and how this might affect motor resonance and social interaction.

In PD progressive loss of dopamine‐producing cells in the substantia nigra, with consequent reduction in innervation of the basal ganglia, results in symptoms including slowness and reduced amplitude of movement. In particular, people with PD have difficulty with the internal generation of actions, in contrast to externally cued actions (Brown & Marsden, 1988; van Wegen, Hirsch, Huiskamp, & Kwakkel, 2014). With the proposed role of the basal ganglia in the action observation network, reduced motor resonance might be expected in PD. Indeed, people with PD have failed to show the modulation of motor evoked potentials during action observation that is found in controls (Tremblay, Leonard, & Tremblay, 2008). Additionally, there is some evidence that processes involving the representation of actions, such as imitation, motor imagery (imagination of movement in the absence of overt action) and action‐related language, may be disrupted in PD (see Poliakoff, 2013; for review). In contrast, other studies have reported intact motor imagery (Heremans *et al*., 2011; van Nuenen *et al*., 2012) and voluntary imitation (Bonivento, Rumiati, Biasutti, & Humphreys, 2013) in PD. Moreover, movement and functional independence in people with PD can be improved through action observation (e.g., Buccino *et al*., 2011) and motor imagery (Kikuchi *et al*., 2014; Tamir, Dickstein, & Huberman, 2007). These apparently contradictory findings might be explained by the involvement of different mechanisms in action representation in PD (Poliakoff, 2013). Data from gesture production (Humphries, Holler, Crawford, Herrera, & Poliakoff, 2016) and body orientation judgement (Conson *et al*., 2014) tasks suggest that people with PD may have particular difficulty in using motor imagery from the first‐person perspective. Neurophysiological studies have shown increased involvement of visual regions such as the extrastriate body area during motor imagery in people with PD, suggesting a greater reliance on visual processes (Helmich, de Lange, Bloem, & Toni, 2007; van Nuenen *et al*., 2012; Wai *et al*., 2012). It is therefore unclear to what extent motor resonance occurs in people with PD, and whether dysfunction of the action observation network may contribute to deficits in social cognition in PD, as proposed by Alegre, Guridi, and Artieda (2011).

This study investigated motor resonance in PD by comparing visuomotor priming for a human hand and a non‐biological stimulus. Although visuomotor priming has previously been investigated in PD, findings are inconclusive. Poliakoff, Galpin, Dick, Moore, and Tipper (2007) compared the effects of observing a task‐irrelevant human finger or a shape (blue square) moving upwards or downwards, while making a finger press or lift response. While stronger compatibility effects for the finger than the shape were found in controls, compatibility effects were equivalent across stimulus types in the PD group. This suggested that motor resonance may be absent or less specific in PD, and is consistent with other findings of reduced specificity in people with PD when responding to visuospatial stimuli (e.g., Praamstra & Plat, 2001). In contrast, Albert, Peiris, Cohen, Miall, and Praamstra (2010) found similar imitative compatibility effects in off‐medication PD patients and controls. Both groups exhibited greater interference in arm movements when observing a human arm, compared to a dot, moving in an incompatible plane. However, in this task, participants were asked to imitate the timing (but not the direction) of the observed action, so attention to the stimuli was greater than in Poliakoff *et al*., where the action stimulus was task‐irrelevant. Thus, it is possible that people with PD only show compatibility effects when attending directly to the movement (Poliakoff, 2013). Importantly, in both of these studies, human movement and non‐biological cues provided the same directional compatibility with the response. The null effect in Poliakoff *et al*. (2007) may therefore have been driven by increased effects of the non‐biological movement in people with PD, which may relate to their heightened responsiveness to simple visual stimuli. Alternatively, the apparent imitative effect in Albert *et al*. (2010) could have been produced by an increased stimulus‐response compatibility effect mediated by increased attention to the human stimulus (see Gowen & Poliakoff, 2012). It therefore remains to be determined whether people with PD exhibit imitative priming for human movement – resulting from motor resonance – as opposed to general stimulus‐response compatibility.

To examine motor resonance in PD, we used a modified visuomotor priming task designed to control for general stimulus‐response compatibility effects that have confounded the interpretation of previous findings. Gowen, Bolton, and Poliakoff (2016) tested visuomotor priming by comparing the effects of a task‐irrelevant moving finger and a non‐biological object (shape) when making a key‐press response to a go signal. In this task, an image of a human hand is rotated by 90 degrees, such that leftward and rightward movements represent upward and downward finger movements respectively. By combining the rotated stimulus with a left‐handed response, imitative compatibility can be dissociated from directional compatibility as well as orthogonal compatibility (right‐up and left‐down pairings; Weeks & Proctor, 1990) and the Simon effect (faster response to stimuli on the same side of space; Simon, 1990). A rightward movement of the stimulus is thus imitatively compatible with the finger press response, while a leftward movement is spatially compatible with the left‐handed response (Simon effect) and the downward movement of the key press (orthogonal compatibility). Gowen *et al*. (2016) found that healthy young adults exhibited general stimulus‐response compatibility for the non‐biological shape (faster responses to leftward movements) immediately following stimulus onset and imitative compatibility effects for the finger (faster responses to rightward movements) at an increased delay. The differences in direction and time course of these effects are consistent with the involvement of different mechanisms in imitative priming and general stimulus‐response compatibility (Catmur & Heyes, 2011).

We predicted that if motor resonance is intact in people with PD, they should exhibit imitative compatibility effects for a human finger movement and general stimulus‐response compatibility effects for a non‐biological shape, and compatibility effects should occur later for the finger than the shape. Moreover, these effects should be of a similar magnitude in people with PD and healthy older controls. In contrast, if previous effects of action observation in PD were driven by low‐level visuospatial processes, we would expect people with PD to exhibit larger stimulus‐response compatibility effects for the shape and smaller or absent imitative compatibility effects for the finger, compared with controls.

## Method

### Participants

Participants with PD were recruited through Parkinson's UK and local neurology clinics. A control group of healthy older adults with no history of neurological illness or injury was recruited from among spouses and partners of participants with PD, as well as through advertising to local community groups. All participants were screened for dementia using the Addenbrooke's Cognitive Examination (ACE‐III; Hsieh, Schubert, Hoon, Mioshi, & Hodges, 2013). Ethical approval for the study was obtained from a UK National Health Service Research Ethics Committee (NRES Committee North West – Liverpool Central), and all participants gave written informed consent.

The PD group consisted of 23 right‐handed participants (eight female) with a mean age of 63.5 years (±6.48; range 47–73), who had a diagnosis of idiopathic PD and presented with mild‐to‐moderate symptoms as indicated by a mean disease stage (Hoehn & Yahr, 1967) of 2.0 (±0.71; range 1–3). The mean time since diagnosis was 6.8 years (±4.79; range 1–20), and participants had a mean score of 38.3 (±11.58; range 16–67; higher scores representing more severe impairment) on the motor examination of the Unified Parkinson's Disease Rating Scale (UPDRS; Goetz *et al*., 2007). All PD participants were tested on their usual medication. The control group consisted of 24 right‐handed participants (13 female) with a mean age of 68.3 (±5.37; range 59–78).

### Procedure

**Figure 1:**
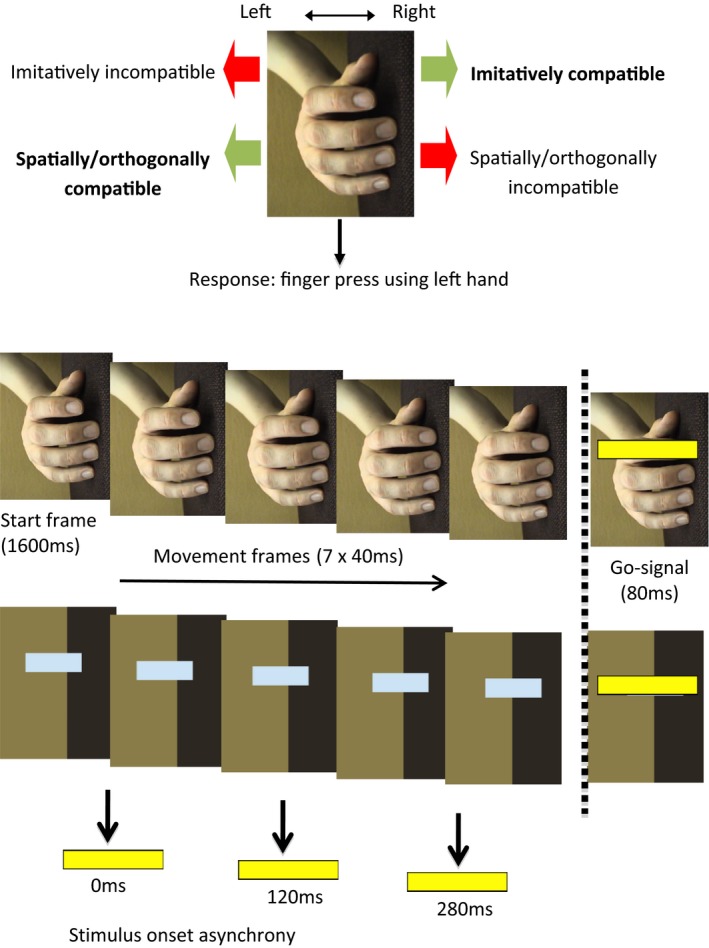
Visuomotor priming task: Compatibility effects and schematic of trial sequence (adapted from Gowen *et al*., 2016). The stimulus (Hand or Shape) begins in a neutral position in the start frame, and then moves for seven frames to the left or right, representing upward or downward movements of the finger respectively. The go signal (yellow flash) is presented at one of three stimulus onset asynchronies (SOAs) following the start frame: 0 ms, 120 ms (after the third frame) or 280 ms (after the final frame; a second end frame is then presented for this SOA). For colour references, please see the online version of this article. [Colour figure can be viewed at http://www.wileyonlinelibrary.com]

Stimuli were displayed using Presentation experimental software (Neurobehavioral Systems, Berkeley, CA, USA). Image sequences (see Figure 1) depicted a human right hand, rotated 90 degrees anticlockwise in a thumb‐up orientation (*Hand* condition), or a blue rectangular shape in an equivalent position and orientation (S*hape* condition). The index finger of the hand, or the shape, moved in a leftward or rightward direction across the screen, corresponding to an upward or downward movement of the finger, respectively. The movement of both the hand and the shape depicted a biological profile, accelerating towards the middle and decelerating towards the end.

The hand stimuli were created by converting digital image (.avi) files into sequences of nine still frames consisting of an initial start frame (finger in a neutral position) seven movement frames and an end frame. The initial frame was presented for 1,600 ms and was identical for upward and downward movements, ensuring that it was not possible to predict the movement direction. The seven movement frames (presented for 40 ms each) depicted the finger making an upward (24.6 mm) or downward (20.2 mm) movement. To plot the trajectory for the Shape condition, a blue rectangle was positioned over the moving index finger in each frame and displayed against a background constructed to resemble that of the hand stimuli (the hand was removed from the image). The luminance (142 cd/m^2^) and size (80 × 25 mm) of the rectangle were matched to the finger shown in the hand stimuli. In test trials, a yellow ‘flash’ (137 × 22 mm) positioned over the finger or shape to cover the full extent of the movement was presented for 80 ms.^1^

Stimuli were presented on a screen at a distance of 1,200 mm from the participant. Participants were required to respond to the appearance of the yellow flash by pressing a key with their left index finger, using a keypad positioned centrally in front of the screen, such that the participant's hand was aligned with the centre of the image.

Participants completed the Shape condition first, followed by the Hand condition.^2^ Figure 1 shows the trial sequence: the moving finger or shape was depicted using the nine frames detailed above, and the go signal (yellow flash) appeared at one of three different stimulus onset asynchronies (SOAs) from the appearance of the first movement frame (0, 120, or 280 ms). The three SOAs were included to assess the strength of priming by the different stimuli at the start, mid‐point and end of the movement. As the 280 ms SOA occurred after the end frame, a second end frame was presented. Test trials were randomly interspersed with no‐go trials (no flash) and baseline trials, in which no movement was seen but the flash appeared after the start frame, following which the start frame was presented again for a further 40 ms. No‐go and baseline trials were included to decrease predictability of the go signal. Trials were terminated when the participant responded, or if no response occurred within 2,000 ms of the appearance of the flash. After each trial, a blank screen was displayed for 2,000 ms. In each block (Hand/Shape), the six types of test trials (compatible/incompatible × 3 SOAs) were presented 12 times, together with 12 baseline and 12 no‐go trials (total = 96 trials per block). There was a short pause halfway through each block to allow the participant to take a rest.

As illustrated in Figure 1, the stimulus orientation and response combination were designed to separate imitative compatibility from more general stimulus‐response compatibility effects: the Simon effect (left side advantage for left‐handed response) and orthogonal spatial compatibility (left advantage for downwards movement). Compatibility was determined by whether the stimulus movement direction was compatible or incompatible with the finger press response. A rightward movement on the screen corresponded to a downward finger movement (imitatively compatible with key press), while a leftward movement corresponded to an upward finger movement (imitatively incompatible with key press). Compatibility effects were calculated by subtracting imitatively compatible from incompatible mean reaction times (RTs). Faster RTs for stimuli moving in the rightward direction than the leftward direction thus result in positive compatibility effects, demonstrating imitative compatibility. In contrast, faster RTs to leftward stimuli than rightward stimuli result in negative compatibility effects, demonstrating non‐imitative stimulus‐response compatibility (Simon effect or orthogonal compatibility).

### Data analysis

Extreme response times (longer than 1,000 ms or shorter than 150 ms) were first excluded; trials outside of 2.25 standard deviations of the participant's mean RT for each stimulus × SOA combination were then removed (van Selst & Jolicoeur, 1994). For the Shape condition, this resulted in the exclusion of 3% of trials in the PD group and 2.9% in the control group. For the Hand condition, 3.1% of trials were excluded in the PD group and 2.7% in the control group. Between‐participant outliers were then identified for each group in each stimulus condition using the same procedure, resulting in the exclusion of data for one participant from each group in the Shape condition and two from each group in the Hand condition.

Compatibility effects were analysed using an ANOVA with stimulus condition, compatibility and SOA as within‐participants factors and group as the between‐participants factor. Non‐parametric (Spearman) correlations between imitative compatibility effects and severity of motor impairment (UPDRS motor examination score) were also analysed for the PD group.

## Results

The groups did not differ significantly in sex, χ^2^(1, 47) = .60; *p* = .44, but the control group were significantly older than the PD group, *t*(45) = 2.80; *p* = .008. However, since age did not correlate significantly with compatibility effects in either group, it was not included as a covariate in the main analyses.

### Compatibility effects

**Figure 2:**
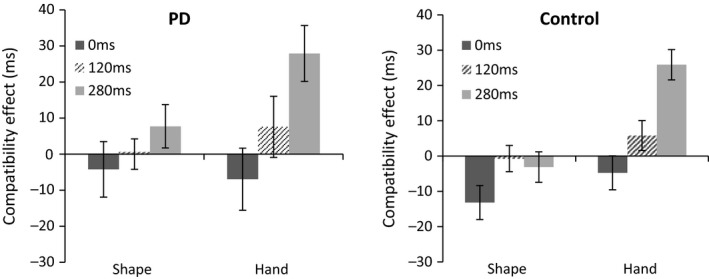
Mean (±1 *SEM*) compatibility effects for Shape and Hand stimuli in each group. Positive values indicate imitative compatibility effects, while negative values indicate general stimulus‐response compatibility.

Compatibility effects are illustrated in Figure 2, with mean RTs for compatible and incompatible trials in Table 1.

**Table 1:** Reaction times and compatibility effects (incompatible – compatible RT) for shape and hand stimuli

Group	Stimulus onset asynchrony (ms)	Shape		Hand	
		Mean RT (ms): Compatible/incompatible	Compatibility effect (±95% confidence interval)	Mean RT (ms): Compatible/incompatible	Compatibility effect (±95% confidence interval)
Parkinson's disease	0	389.70	−4.22 (15.64)	394.86	−5.41 (9.52)
		385.48		389.45	
	120	383.87	0.01 (8.62)	383.36	10.64 (14.74)
		383.87		394.00	
	280	385.43	7.74 (12.21)	381.24	28.34 (19.68)
		393.17		409.57	
Control	0	366.14	−13.12 (10.03)	368.04	−4.77 (11.61)
		352.95		363.27	
	120	361.70	−0.72 (7.67)	357.23	5.81 (14.24)
		360.98		363.03	
	280	363.03	−3.14 (8.99)	362.11	25.87 (14.26)
		359.89		387.99	

There was a significant main effect of compatibility (*F*(1, 45) = 4.69; *p* = .036; η^2^ *p* = .11), with shorter RTs for compatible than incompatible trials (mean difference = 4.16 ms). The effect of stimulus was marginally significant (*F*(1, 45) = 3.57; *p* = .066; η^2^ *p* = .08), reflecting shorter overall RTs to the shape than the hand (mean difference = 10.30 ms). There were significant interactions between stimulus and compatibility (*F*(1, 45) = 7.82; *p* = .008; η^2^ *p* = .17), and between compatibility and SOA (*F*(2, 90) = 15.04; *p* < .001; η^2^ *p* = .28), with a significant three‐way interaction between stimulus, compatibility and SOA (*F*(2, 90) = 4.06; *p* = .021; η^2^ *p* = .09). Bonferroni corrected *t*‐tests revealed that compatibility effects were significantly greater for the hand than the shape at 280 ms, mean difference = 25.98; *t*(42) = 3.91; *p* < .001; *d* = 0.61*,* but did not differ at 0 ms, mean difference = 4.10; *t*(40) = .60; *p* > .01; *d* = 0.09, or 120 ms, mean difference = 7.45; *t*(40) = 1.29; *p* = .60; *d* = 0.21. Analysis of simple effects showed a significant positive compatibility effect for the hand at 280 ms, *t*(42) = 4.73; *p* < .001; *d* = 0.72, but not at 0 ms, *t*(42) = −1.42; *p* = .48; *d* = 0.22*,* or 120 ms, *t*(42) = 1.68; *p* = .30; *d* = 0.26, while compatibility effects for the shape were not significant at any of the SOAs: 0 ms, *t*(44) = −1.99; *p* = .16; *d* = 0.30, 120 ms, *t*(44) = −.13; *p* > .1; *d* = 0.02, or 280 ms, *t*(44) = .59; *p* > .1; *d* = 0.09. The effect of SOA (*F*(2, 90) = 1.71; *p* = .19; η^2^ *p* = .042), and the interaction between SOA and stimulus (*F*(2, 90) = 1.52; *p* = .23; η^2^ *p* = .037), were not significant.

There was no significant overall difference in RTs between groups (*F*(1, 45) = 1.91; *p* = .18; η^2^ *p* = .047), and there were no significant interactions of group with stimulus (*F*(1, 45) = .006; *p* = .94; η^2^ *p* < .001), compatibility (*F*(1, 45) = .35; *p* = .56; η^2^ *p* = .012), or SOA (*F*(2, 90) = .12; *p* = .89; η^2^ *p* = .003). Interactions were not significant between group, stimulus and compatibility (*F*(1, 45) = .48; *p* = .49; η^2^ *p* = .01), group, stimulus and SOA (*F*(2, 90) = .62; *p* = .54; η^2^ *p* = .016), group, compatibility and SOA (*F*(2, 90) = .051; *p* = .95; η^2^ *p* = .001) or group, stimulus, compatibility and SOA (*F*(2, 90) = .14; *p* = .87; η^2^ *p* = .004).

### Relationship with disease severity

In the PD group, the UPDRS motor score correlated positively with overall compatibility effects for the hand, *r*(20) = .58; *p* = .005, but not the shape, *r*(20) = −.05; *p* = .82, associating increasing disease severity with larger imitative compatibility effects (greater difference in RTs between compatible and incompatible trials). Further analysis of RTs for the hand stimulus at 280 ms (where a significant compatibility effect was found) revealed a significant correlation for incompatible trials, *r*(19) = .47; *p* = .031, but not compatible trials, *r*(19) = .27; *p* = .24, indicating that participants with more severe motor impairment were slowed down more by observing incompatible movements.

## Discussion

The present study investigated motor resonance in PD by examining imitative compatibility effects for observed actions. In previous studies of visuomotor priming in PD (Albert *et al*., 2010; Poliakoff *et al*., 2007), true imitative compatibility was not dissociated from more general stimulus‐response compatibility effects. Using a task designed to separate out these effects, we found imitative compatibility for a human hand that was of a similar magnitude in people with PD and controls, indicating intact motor resonance. Furthermore, these findings also provide the first clear evidence of imitative priming in healthy older adults, which has been similarly confounded by general stimulus‐response compatibility in previous studies (Albert *et al*., 2010; Poliakoff *et al*., 2007; Verrel, Lisofsky, Kuhn, & Lindenberger, 2016).

Two further findings of this study are indicative of preserved motor resonance. First, we found a tendency for slower overall responses for the hand than for a moving non‐biological shape, consistent with findings from young participants, and suggesting that motor simulation may have been involved (Gowen *et al*., 2016). Second, imitative compatibility effects for the hand were only observed at the longest stimulus onset asynchrony. This is consistent with previous observations that imitative compatibility effects tend to increase over time following stimulus presentation, while general stimulus‐response effects tend to decrease (Brass *et al*., 2001; Catmur & Heyes, 2011). Unlike Gowen *et al*. (2016), we did not find significant effects of stimulus‐response compatibility for the shape, in either the PD group or the control group. It is unclear why this stimulus‐response compatibility was found in younger adults but not older adults with or without PD, but it may be related to more variable response times or a general effect of ageing on attention, such as slower orienting to visual stimuli (see Erel & Levy, 2016). Nevertheless, previous studies have demonstrated spatial compatibility effects in PD for moving shapes (Albert *et al*., 2010; Poliakoff *et al*., 2007).

The present results extend previous findings of imitative compatibility effects in PD (Albert *et al*., 2010), by demonstrating that imitative compatibility occurs independently of general stimulus‐response compatibility effects, and with observation of a task‐irrelevant action. In contrast to Poliakoff *et al*. (2007), our results showed that people with PD responded differentially to a moving shape and a human finger. However, this previous study was not able to isolate imitative compatibility, and the late onset of the go signal (440 ms) meant that compatibility effects were small in magnitude. Our findings are also consistent with evidence indicating that people with PD can effectively use motor imagery, such as when judging the weight of objects after viewing a lifting action (Poliakoff, Galpin, Dick, & Tipper, 2010), or in the production of appropriate gestures when communicating action‐related information (Humphries *et al*., 2016). Moreover, the present findings provide a clear rationale for therapies that capitalize on intact motor resonance, consistent with emerging evidence supporting the application of action observation training in PD (for recent reviews see Abbruzzese, Avanzino, Marchese, & Pelosin, 2015; Caligiore, Mustile, Spalletta, & Baldassarre, 2017). Nonetheless, as described above, people with PD may engage compensatory visual processes during motor imagery (Helmich *et al*., 2007; van Nuenen *et al*., 2012; Wai *et al*., 2012) as well as showing reduced corticomotor facilitation when observing actions (Tremblay *et al*., 2008). Despite similar performance between groups in the visuomotor priming task, the underlying mechanisms may thus be different in people with PD, and the generation of action representations for motor imagery or overt action may still be compromised, as reflected in the impairment of internally generated action (e.g., D'Andrea, Haffenden, Furtado, Suchowersky, & Goodyear, 2013). However, our results suggest that action observation may provide an effective external cue to trigger this process (see also Poliakoff, 2013).

It is also important to consider whether the effects of observing actions differ from those of observing affordances provided by action‐relevant objects. Galpin, Tipper, Dick, and Poliakoff (2011) found that, in people with PD, compatibility effects from action‐relevant door handles did not differ from those produced by a simple bar. When compared to the present findings, this suggests that observing human movement may have a stronger influence than objects on action in PD, particularly when it provides specific action‐relevant information (in this case, priming the finger press action). Nevertheless, action‐relevant objects do influence movement in PD (see, Poliakoff, 2013 for a review). Therefore, it would be relevant to explore the different motor effects afforded by human movement and objects in PD, particularly when viewing multiple object displays (where it is hypothesized that people with PD will be affected; Caligiore *et al*., 2013), as well as the effects of distractor actions. Moreover, investigations of compatibility effects for object‐directed actions could inform the design of action observation‐based therapies targeting functional movements.

Our findings also have implications for understanding social cognition deficits that can occur in PD (e.g., Elamin, Pender, Hardiman, & Abrahams, 2012; Narme *et al*., 2013). In particular*,* it has been proposed that impaired social‐perceptive processing in PD may be related to dysfunction of the mirror neuron system (Alegre *et al*., 2011); however, our results suggest that activation of this system in people with mild‐to‐moderate PD is similar to that of healthy older adults. Additionally, people with PD report engaging in behavioural mimicry in social situations (e.g., mirroring a partner's hand gestures; Bek *et al*., 2016), and show appropriate spontaneous facial expressions in response to emotional and social context (Simons, Pasqualini, Reddy, & Wood, 2004). Nevertheless, because of a general reduction in spontaneous actions, overt signs of motor resonance may be less apparent in PD.

The magnitude of imitative compatibility effects in the PD group was related to the severity of motor signs, with greater interference from incompatible stimuli associated with increasing motor impairment. Thus, individuals who are physically slower to respond may have more opportunity to be influenced by incompatible stimuli. As people with PD can have difficulty with inhibition and impulse control (e.g., Wylie *et al*., 2012), it is also possible that those who are more severely affected are less able to inhibit the processing of observed actions.

The ability to inhibit imitation is required in order to respond appropriately according to social context, and imitative control has been linked to social cognition and perspective taking (Santiesteban *et al*., 2012; Spengler, Bird, & Brass, 2010). If imitative control declines with increasing disease severity in PD, this may impact upon social interaction and understanding. Therefore, further investigation of the relationships between motor resonance, inhibition and social cognition in PD would be informative.

### Conclusions

People with PD and healthy older adults show imitative priming elicited by observed hand actions. Importantly, we have demonstrated for the first time that this cannot be accounted for by general stimulus‐response compatibility effects. These findings support the use of action observation in neurorehabilitation.

## Supporting information


**Appendix S1.** Motor resonance in Parkinson's disease.

## Footnotes

1 It should be noted that in this study, stimuli were displayed on a projector screen for consistency with other experimental tasks conducted with this group; as such, stimuli were approximately 40% larger than those used in Gowen *et al*. (2016), although participants were also seated at a greater distance from the screen (1,200 mm vs. 800 mm).

2 Based on findings of top‐down effects on automatic imitation in young healthy adults (Gowen *et al*., 2016), the Shape block was repeated (before the Hand condition) after participants were informed that the shape represented a moving human finger. However, compatibility effects for the shape were not significant either before or after the belief manipulation and did not differ between blocks. Results of this analysis are presented in the Supporting Information.
