# Supplementary material for: Action observation produces motor resonance in Parkinson's disease
Source: J Neuropsychol. 2017 Sep 11;12(2):298–311. doi: 10.1111/jnp.12133 (PMC6001452; doi:10.1111/jnp.12133)
Supplement: Supplementary file 1 — Appendix S1. Motor resonance in Parkinson's disease. [file JNP-12-298-s001.docx]

**Action observation produces motor resonance in Parkinson’s disease**

**Supplementary material**

Based on findings from young healthy adults (Gowen et al., 2016), we explored top-down effects on visuomotor priming in PD and healthy ageing using a belief manipulation. After completing the Shape block, participants were informed that the shape in fact represented a moving human finger (this was also demonstrated using an illustration showing how the hand had been rotated); the Shape block was then repeated, prior to the Hand condition.

Data were analysed using an ANOVA with compatibility (compatible, incompatible), stimulus condition (Shape, Belief, Hand), and SOA (0 ms, 120 ms, 280 ms) as within-participants factors and group as the between-participants factor. Extreme response times (longer than 1000 ms or shorter than 150 ms) were first excluded; trials outside of 2.25 standard deviations of the participant’s mean RT for each stimulus x SOA combination were then removed. This resulted in the exclusion of 3% of trials in the PD group and 2.9% in the control group for the Shape condition, 3.4% of trials in the PD group and 3.3% in the control group for the Belief condition, and 3.1% of trials in the PD group and 2.7% in the control group for the Hand condition. Between-participant outliers were then identified, resulting in the exclusion of data for one participant from each group in the Shape condition, two from each group in the Hand condition, and one from the PD group in the Belief condition.

Compatibility effects are illustrated in Figure 1, with mean RTs for compatible and incompatible trials in Table 1. Main effects of compatibility (F(1,45) = .44; P = .51; η^2^p = .012), stimulus condition (F(2,90) = 1.56; P = .22; η^2^p = .039) and SOA (F(2,90) = 1.06; P = .35; η^2^p = .027) were not significant. There were significant interactions between stimulus condition and compatibility (F(2,90) = 6.71; P = .002; η^2^p = .15), compatibility and SOA (F(2,90) = 14.37; P < .001; η^2^p = .27), and stimulus and SOA (F(2, 90) = 6.99; P < .001; η^2^p = .16). There was also a significant three-way interaction between stimulus, compatibility and SOA (F(4, 180) = 3.10; P = .017; η^2^p = .075): compatibility effects were significantly greater for Hand than Shape (mean difference = 25.98; t(42) = 3.91; P < .001; *d =.61*) and Belief (mean difference = 28.40; t(42) = 3.90; P < .001; *d =.60*) at 280 ms. Compatibility effects for Hand did not differ from Shape at 0 ms (mean difference = 4.10; t(40) = .60; P >.1; *d =.09.*) or 120 ms (mean difference = 7.45; t(40) = 1.29; P = .60; *d =.21*), or from the Belief condition at 0 ms (mean difference = 4.83; t(41) = .85; P >.1; *d =.13*) or 120 ms (mean difference = 5.82; t(41) = .86; P >.1; *d =.13*). The Shape and Belief conditions did not differ at 0 ms (mean difference = -3.87; t(42) = -.51; P >.1; *d =.08*), 120 ms (mean difference = .18; t(42) = .04; P >.1; *d =.006*) or 280 ms (mean difference = -2.79; t(42) = -.60; P >.1; *d =.09*). Simple effects analysis revealed a significant positive compatibility effect at 280 ms for the hand (t(42) = 4.73; P < .001; *d =.72*). All other compatibility effects were non-significant. The interaction between stimulus and SOA was not significant (F(2,90) = .89; P = .42; *η^2^p* = .019).

There was no significant effect of group (F(1,45) = 1.85; p = .18; *η^2^p* = .046), and there were no significant interactions of group with stimulus condition (F(1,90) = 1.53; P = .22; *η^2^p* = .039), compatibility (F(1, 45) = .33; P = .57; *η^2^p* = .009) or SOA (F(1, 90) = .56; P = .58; *η^2^p* = .014). There were no significant interactions between group, stimulus and compatibility (F(1,90) = .59; P = .56; *η^2^p* = .019), group, stimulus and SOA (F(4,180) = 1.38; P = .24; *η^2^p* = .035), group, compatibility and SOA (F(2, 90) = .96; P = .39; *η^2^p* = .025), or group, stimulus, compatibility and SOA (F(4,180) = 1.48; P = .21; *η^2^p* = .038).

*[Figure 1 here]*

**Discussion**

People with PD and healthy older adults exhibited imitative compatibility effects when observing a moving human hand. Compatibility effects for a non-biological shape were not influenced by a belief manipulation in which participants were informed that the shape represented a moving finger. These results indicate that neither people with PD nor healthy older adults represented the shape in terms of human movement.

Our findings contrast with those of Gowen et al. (2016), using a similar task in healthy young adults. Gowen et al. found imitative compatibility effects for the shape stimulus following a belief manipulation, which were similar to that for the hand. Similar response times in the hand and belief conditions further indicated the engagement of action representation mechanisms following the belief manipulation. The absence of a belief effect in both individuals with PD and healthy older participants in the present study may reflect a general decline in top-down control of action representation with age. However, previous findings on belief effects in visuomotor priming studies have been inconsistent (Gowen & Poliakoff, 2012), and further research is needed to explore effects of belief and other top-down factors on action representation in healthy ageing and neurological conditions.

**Figure 1**


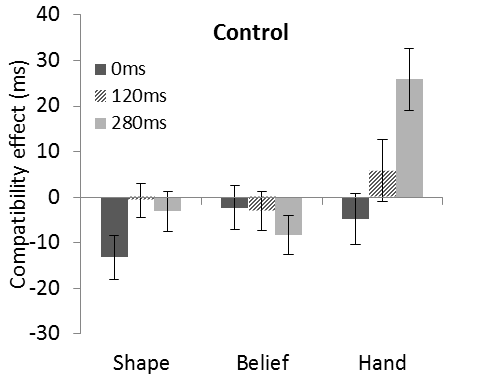

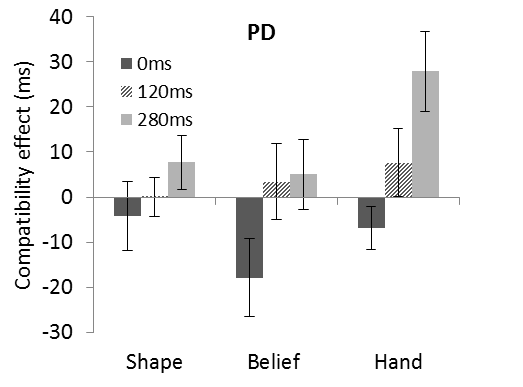


Figure 1. Mean (±1SEM) compatibility effects for Shape, Belief and Hand conditions. Positive values indicate imitative compatibility effects, while negative values indicate general stimulus-response compatibility.

|  |  | Shape | | Belief | | Hand | |
| --- | --- | --- | --- | --- | --- | --- | --- |
| Group | SOA (ms) | Mean RT (ms):  Compatible/ incompatible | Compatibility effect (±95% confidence interval) | Mean RT (ms):  Compatible/ incompatible | Compatibility effect (±95% confidence interval) | Mean RT (ms):  Compatible/ incompatible | Compatibility effect (±95% confidence interval) |
| PD | 0 | 389.70 | -4.22 (15.64) | 423.87 | -17.93 (17.53) | 394.86 | -5.41 (9.52) |
|  |  | 385.48 |  | 405.94 |  | 389.45 |  |
|  | 120 | 383.87 | .01 (8.62) | 398.42 | 3.38 (17.27) | 383.36 | 10.64 (14.74) |
|  |  | 383.87 |  | 401.80 |  | 394.00 |  |
|  | 280 | 385.43 | 7.74 (12.21) | 393.40 | 5.02 (15.76) | 381.24 | 28.34 (19.68) |
|  |  | 393.17 |  | 398.42 |  | 409.57 |  |
| Control | 0 | 366.14 | -13.12 (10.03) | 364.85 | -2.34 (9.92) | 368.04 | -4.77 (11.61) |
|  |  | 352.95 |  | 362.51 |  | 363.27 |  |
|  | 120 | 361.70 | -.72 (7.67) | 365.83 | -3.00 (8.74) | 357.23 | 5.81 (14.24) |
|  |  | 360.98 |  | 362.83 |  | 363.03 |  |
|  | 280 | 363.03 | -3.14 (8.99) | 359.13 | -8.21 (8.83) | 362.11 | 25.87 (14.26) |
|  |  | 359.89 |  | 350.92 |  | 387.99 |  |

Table 1. Reaction times and compatibility effects (incompatible – compatible RT) for Shape, Belief and Hand conditions.
